# Novel genomic resources for shelled pteropods: a draft genome and target capture probes for *Limacina bulimoides*, tested for cross-species relevance

**DOI:** 10.1186/s12864-019-6372-z

**Published:** 2020-01-03

**Authors:** Le Qin Choo, Thijs M. P. Bal, Marvin Choquet, Irina Smolina, Paula Ramos-Silva, Ferdinand Marlétaz, Martina Kopp, Galice Hoarau, Katja T. C. A. Peijnenburg

**Affiliations:** 1 Marine Biodiversity, Naturalis Biodiversity Center, Leiden, The Netherlands; 2 Institute for Biodiversity and Ecosystem Dynamics (IBED), University of Amsterdam, Amsterdam, The Netherlands; 3 Faculty of Biosciences and Aquaculture, Nord University, Bodø, Norway; 4 Molecular Genetics Unit, Okinawa Institute of Science and Technology, Onna-son, Japan

**Keywords:** Targeted sequencing, Exon capture, Genome, Non-model organism, Marine zooplankton

## Abstract

**Background:**

Pteropods are planktonic gastropods that are considered as bio-indicators to monitor impacts of ocean acidification on marine ecosystems. In order to gain insight into their adaptive potential to future environmental changes, it is critical to use adequate molecular tools to delimit species and population boundaries and to assess their genetic connectivity. We developed a set of target capture probes to investigate genetic variation across their large-sized genome using a population genomics approach. Target capture is less limited by DNA amount and quality than other genome-reduced representation protocols, and has the potential for application on closely related species based on probes designed from one species.

**Results:**

We generated the first draft genome of a pteropod, *Limacina bulimoides*, resulting in a fragmented assembly of 2.9 Gbp. Using this assembly and a transcriptome as a reference, we designed a set of 2899 genome-wide target capture probes for *L. bulimoides*. The set of probes includes 2812 single copy nuclear targets, the 28S rDNA sequence, ten mitochondrial genes, 35 candidate biomineralisation genes, and 41 non-coding regions. The capture reaction performed with these probes was highly efficient with 97% of the targets recovered on the focal species. A total of 137,938 single nucleotide polymorphism markers were obtained from the captured sequences across a test panel of nine individuals. The probes set was also tested on four related species: *L. trochiformis*, *L. lesueurii*, *L. helicina*, and *Heliconoides inflatus*, showing an exponential decrease in capture efficiency with increased genetic distance from the focal species. Sixty-two targets were sufficiently conserved to be recovered consistently across all five species.

**Conclusion:**

The target capture protocol used in this study was effective in capturing genome-wide variation in the focal species *L. bulimoides*, suitable for population genomic analyses, while providing insights into conserved genomic regions in related species. The present study provides new genomic resources for pteropods and supports the use of target capture-based protocols to efficiently characterise genomic variation in small non-model organisms with large genomes.

## Background

Shelled pteropods are marine, holoplanktonic gastropods commonly known as ‘sea butterflies’, with body size ranging from a few millimetres (most species) to 1–2 cm [1]. They constitute an important part of the global marine zooplankton assemblage e.g. [2, 3] and are a dominant component of the zooplankton biomass in polar regions [4, 5]. Pteropods are also a key functional group in marine biogeochemical models because of their high abundance and dual role as planktonic consumers as well as calcifiers e.g. [6, 7]. Shelled pteropods are highly sensitive to dissolution under decreasing oceanic pH levels [2, 8, 9] because their shells are made of aragonite, an easily soluble form of calcium carbonate [10]. Hence, shelled pteropods may be the ‘canaries in an oceanic coal mine’, signalling the early effects of ocean acidification on marine organisms caused by anthropogenic releases of CO_2_ [5, 11]. In spite of their vulnerability to ocean acidification and their important trophic and biogeochemical roles in the global marine ecosystem, little is known about their resilience towards changing conditions [5].

Given the large population sizes of marine zooplankton in general, including shelled pteropods, adaptive responses to even weak selective forces may be expected as the loss of variation due to genetic drift should be negligible [12]. Furthermore, the geographic scale over which gene flow occurs, between populations facing different environmental conditions, may influence their evolutionary potential [13] and consequently needs to be accounted for. It is thus crucial to use adequate molecular tools to delimit species and population boundaries in shelled pteropods.

So far, genetic connectivity studies in shelled pteropods have been limited to the use of single molecular markers. Analyses using the mitochondrial cytochrome oxidase subunit I (COI) and the nuclear 28S genes have revealed dispersal barriers at basin-wide scales in pteropod species belonging to the genera *Cuvierina* and *Diacavolinia* [14, 15]. For *Limacina helicina*, the Arctic and Antarctic populations were discovered to be separate species through differences in the COI gene [16, 17]. However, the use of a few molecular markers has often been insufficient to detect subtle patterns of population structure expected in high gene flow species such as marine fish and zooplankton [18–20]. In order to identify potential barriers to dispersal, we need to sample a large number of loci across the genome, which is possible due to recent developments in next-generation sequencing (NGS) technologies [21, 22].

Here, we chose a genome reduced-representation method to characterise genome-wide variation in pteropods because of their potentially large genome sizes and small amount of input DNA per individual. In species with large genomes, as reported for several zooplankton groups [20], whole genome sequencing may not be feasible for population-level studies. Reduced-representation methods can overcome the difficulty of sequencing numerous large genomes. Two common approaches are RADseq and target capture enrichment. RADseq [23], which involves the enzymatic fragmentation of genomic DNA followed by the selective sequencing of the regions flanking the restriction sites of the used enzyme(s), is attractive for non-model organisms as no prior knowledge of the genome is required. However, RADseq protocols require between 50 ng and 1 μg of high-quality DNA, with higher amounts being recommended for better performance [24], and has faced substantial challenges in other planktonic organisms e.g. [25, 26]. Furthermore, RADseq may not be cost efficient for species with large genomes [26]. Target capture enrichment [27–29] overcomes this limitation in DNA starting amount and quality, by using single-stranded DNA probes to selectively hybridise to specific genomic regions that are then recovered and sequenced [30]. It has been successfully tested on large genomes with just 10 ng of input DNA [31] as well as degraded DNA from museum specimens [32–35]. Additionally, the high sequencing coverage of targeted regions allows rare alleles to be detected [31].

Prior knowledge of the genome is required for probe design, however, this information is usually limited for non-model organisms. Currently, there is no pteropod genome available that can be used for the design of genome-wide target capture probes. The closest genome available is from the sister group of pteropods, Anaspidea (*Aplysia californica* (NCBI reference: PRJNA13635) [36]), but it is too distant to be a reference, as pteropods have diverged from other gastropods since at least the Late Cretaceous [37].

In this study, we designed target capture probes for the shelled pteropod *Limacina bulimoides* based on the method developed in Choquet et al. [26], to address population genomic questions using a genome-wide approach. We obtained the draft genome of *L. bulimoides* to develop a set of target capture probes, and tested the success of these probes through the number of single nucleotide polymorphisms (SNPs) recovered in the focal species. *L. bulimoides* was chosen as the probe-design species because it is an abundant species with a worldwide distribution across environmental gradients in subtropical and tropical oceans. The probes were also tested on four related species within the Limacinoidea superfamily (coiled-shell pteropods) to assess their cross-species effectiveness. Limacinoid pteropods have a high abundance and biomass in the world’s oceans [2, 6, 37] and have been the focus of most ocean acidification research to date e.g. [2, 38, 39].

## Results

### Draft genome assembly

We obtained a draft genome of *L. bulimoides* (NCBI: SWLX00000000) from 108 Gb of Illumina data sequenced as 357 million pairs of 150 base pair (bp) reads. As a first pass in assessing genomic data completeness, a k-mer spectrum analysis was done with JELLYFISH version 1.1.11 [40]. It did not show a clear coverage peak, making it difficult to estimate total genome size with the available sequencing data (Additional file 1: Appendix S1). Because distinguishing sequencing error from a coverage peak is difficult below 10-15x coverage, it is likely that the genome coverage is below 10-15x, suggesting a genome size of at least 6–7 Gb. The reads were assembled using the de novo assembler MaSuRCA [41] into 3.86 million contigs with a total assembly size of 2.9 Gbp (N50 = 851 bp, L50 = 1,059,429 contigs). The contigs were further assembled into 3.7 million scaffolds with a GC content of 34.08% (Table 1). Scaffolding resulted in a slight improvement, with an increase in the N50 to 893 bp and a decrease in the L50 to 994,289 contigs. Based on the hash of error corrected reads in MaSuRCA, the total haploid genome size was estimated at 4,801,432,459 bp (4.8 Gbp). Therefore, a predicted 60.4% of the complete genome was sequenced.

**Table 1:** Summary of draft genome statistics for *Limacina bulimoides*

Assembly statistics	Value
Estimated total genome size	4,801,432,559 bp
Total assembly size	2,901,932,435 bp
Number of scaffolds	
> = 0 bp	3,735,734
> = 1000 bp	802,059
> = 5000 bp	3890
> = 10,000 bp	116
> = 25,000 bp	6
> = 50,000 bp	3
N50	893 bp
L50	994,289
Smallest scaffold	200 bp
Largest scaffold	197,255 bp
Percentage of N’s	0.3307
GC content, %	34.08

Genome completeness based on the assembled draft genome was measured in BUSCO version 3.0.1 [42] and resulted in the detection of 60.2% of near universal orthologues that were either completely or partially present in the draft genome of *L. bulimoides* (Table 2). This suggests that around 40% of gene information is missing or may be too divergent from the BUSCO sets [42]. Although the use of BUSCO on a fragmented genome may not give reliable estimates as orthologues may be partially represented within scaffolds that are too short for a positive gene prediction, this percentage of near-universal orthologues coincides with the estimate of genome size by MaSuRCA.

**Table 2:** Summary of BUSCO analysis showing the number of metazoan near universal orthologues that could be detected in the draft genome of *Limacina bulimoides*

	Present in draft genome
Complete	296 (30.3%)
Complete and single-copy	262 (26.8%)
Complete and duplicated	34 (3.5%)
Fragmented	292 (29.9%)
Missing	390 (39.8%)
Total BUSCO groups searched	978

We also compared the draft genome to a previously generated transcriptome of *L. bulimoides* (NCBI: SRR10527256) [43] to assess the completeness of the coding sequences and aid in the design of capture probes. The transcriptome consisted of 116,995 transcripts, with an N50 of 555 bp. Even though only ~ 60% of the genome was assembled, 79.8% (93,306) of the transcripts could be mapped onto it using the splice-aware mapper GMAP version 2017-05-03 [44]. About half of the transcripts (46,701 transcripts) had single mapping paths and the other half (46,605 transcripts) had multiple mapping paths. These multiple mapping paths are most likely due to the fragmentation of genes over at least two different scaffolds, but may also indicate multi-copy genes or transcripts with multiple spliced isoforms. Of the singly mapped transcripts, 8374 mapped to a scaffold that contained two or more distinct exons separated by introns. Across all the mapped transcripts, 73,719 were highly reliable with an identity score of 95% or higher.

### Target capture probes design and efficiency

**Fig. 1:**
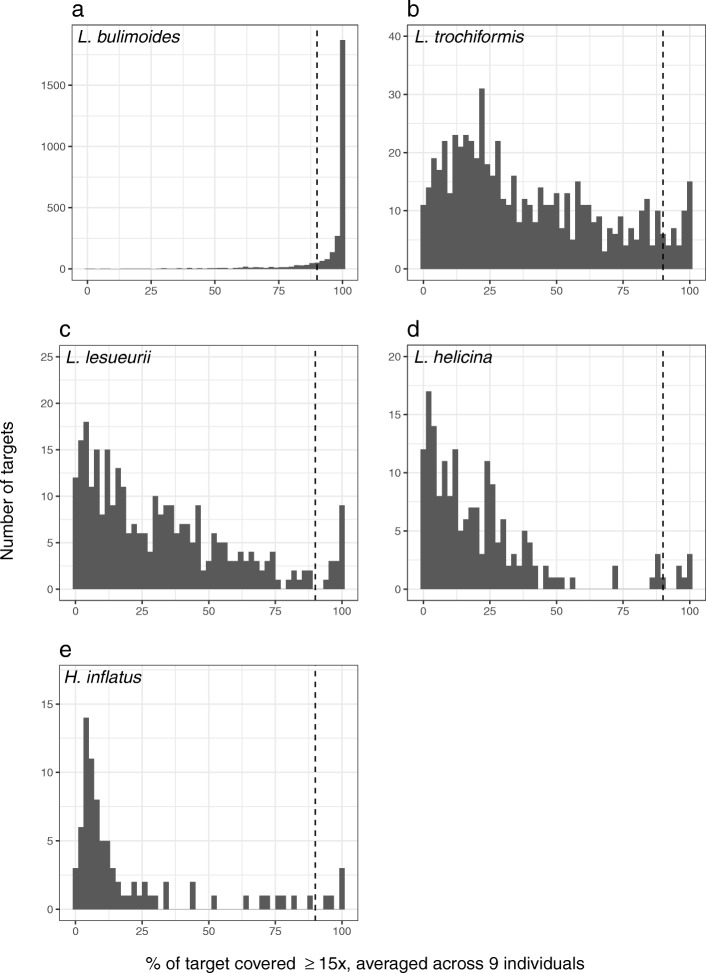
Number of recovered targets plotted against average proportion of bases in each target, with at least 15x sequencing coverage averaged across nine individuals, for each for the five shelled pteropod species (**a**: *Limacina bulimoides*, **b**: *L. trochiformis*, **c**: *L. lesueurii*, **d**: *L. helicina*, and **e**: *Heliconoides inflatus*). Bars on the right of the dashed vertical line represent the number of targets where more than 90% of the bases in each target was sequenced with ≥15x depth. Note the differences in y-axes between the plots. There is no peak at one SNP for *L. bulimoides* (Additional file 1: Appendix S5)

A set of 2899 genome-wide probes, ranging from 105 to 1095 bp, was designed for *L. bulimoides*. This includes 2812 single copy nuclear targets of which 643 targets were previously identifed as conserved pteropod orthologs [43], the 28S rDNA sequence, 10 known mitochondrial genes, 35 candidate biomineralisation genes [45, 46], and 41 randomly selected non-coding regions (see Methods). The set of probes worked very well on the focal species *L. bulimoides*. 97% (2822 of 2899 targets) of the targeted regions were recovered across a test panel of nine individuals (Table 3) with 137,938 SNPs (Table 4) identified across these targeted regions. Each SNP was present in at least 80% of *L. bulimoides* individuals (also referred to as genotyping rate) with a minimum read depth of 5x. Coverage was sufficiently high for SNP calling (Fig. 3) and 87% of the recovered targets (2446 of the 2822 targets) had a sequence depth of 15x or more across at least 90% of their bases (Fig. 1a). Of the 2822 targets, 643 targets accounted for 50% of the total aligned reads in *L. bulimoides* (Additional file 1: Figure S2A in Appendix S2). For *L. bulimoides*, SNPs were found in all categories of targets, including candidate biomineralisation genes, non-coding regions, conserved pteropod orthologues, nuclear 28S and other coding sequences (Table 5). Of the 10 mitochondrial genes included in the capture, surprisingly, only the COI target was recovered.

**Table 3:** Target capture efficiency statistics, averaged ± standard deviation across nine individuals, for each of five pteropod species, including raw reads, final mapped reads, % High Quality reads (reads mapping uniquely to the targets with proper pairs), % targets covered (percentage of bases across all targets covered by at least one read), average depth (sequencing depth across all targets with reads mapped)

Species	Raw reads (× 1,000)	Final mapped reads (× 1,000)	% HQ reads	% targets covered	Average depth
*L. bulimoides*	10,529 ± 3997	3531 ± 1548	33.23 ± 9.10	97.36 ± 0.42	250 ± 111
*L. trochiformis*	15,508 ± 4865	1765 ± 521	11.61 ± 2.59	20.32 ± 1.65	468 ± 144
*L. lesueurii*	7060 ± 2043	807 ± 196	11.93 ± 2.77	13.28 ± 1.96	431 ± 76.9
*L. helicina*	10,346 ± 6260	337 ± 180	3.47 ± 0.56	12.57 ± 2.71	63.7 ± 26.7
*H. inflatus*	3089 ± 1126	66 ± 30	2.07 ± 0.30	8.21 ± 3.34	31.9 ± 14.9

**Table 4:** Number of single nucleotide polymorphism (SNPs) recovered after various filtering stages for five species of shelled pteropods. Hard-filtering was implemented in GATK3.8 VariantFiltration using the following settings: QualByDepth <2.0, FisherStrand >60.0, RMSMappingQuality <5.0, MQRankSumTest <-5.0 and ReadPositionRankSum <-5.0. The hard-filtered SNPs were subsequently filtered to keep those with a minimum site coverage of 5x and present in at least 80% of the individuals. Other filtering options were less stringent, such as a minimum depth of 2x and site presence in at least 50% of individuals

	Hard-filtering	80% individuals, 5x depth	80% individuals, 2x depth	50% individuals,5x depth
*L. bulimoides*	154,864	137,938	137,953	147,763
*L. trochiformis*	44,014	11,948	12,165	20,518
*L. lesueurii*	23,379	5359	5847	8487
*L. helicina*	18,298	2432	2771	4613
*H. inflatus*	13,041	1371	1559	2092

**Table 5:** Number of targets with at least one single nucleotide polymorphism (based on 80% genotyping rate, 5x depth) was calculated according to category: candidate biomineralisation genes (Biomin.), conserved pteropod orthologues (Ortholog.), mitochondrial (Mt genes), nuclear 28S, and other coding and non-coding regions for each of five pteropod species. Numbers in brackets represent the total number of targets in that category on the set of target probes designed for *Limacina bulimoides*

Species	Biomin. (35)	Ortholog. (643)	Mt genes (10)	28S (1)	Coding (2169)	Non-coding (41)	Total (2899)
*L. bulimoides*	32	635	1	1	2140	13	2822
*L. trochiformis*	7	169	3	1	436	4	620
*L. lesueurii*	0	90	2	1	209	0	302
*L. helicina*	0	52	3	1	121	0	177
*H. inflatus*	0	20	1	1	61	0	83

The hybridisation of the probes and targeted re-sequencing worked much less efficiently on the four related species. The percentage of targets covered by sequenced reads ranged from 8.21% (83 out of 2899 targets) in *H. inflatus* to 20.32% (620 out of 2899 targets) in *L. trochiformis* (Table 3). Of these, only five (*H. inflatus*) to 42 (*L. trochiformis*) targets were covered with a minimum of 15x depth across 90% of the bases (Additional file 1: Table S1). The number of targets that accounted for 50% of the total aligned reads varied across species, with 4 of 620 targets for *L. trochiformis* that accounted for 50% of reads, 2 of 302 targets for *L. lesueurii*, 14 of 177 targets for *L. helicina* and 5 of 83 targets for *H. inflatus* (Additional file 1: Figure S2B-E in Appendix S2). In these four species, targeted regions corresponding to the nuclear 28S gene, conserved pteropod orthologues, mitochondrial genes and other coding sequences were obtained (Table 4). The number of mitochondrial targets recovered ranged between one and three: ATP6, COB, 16S were obtained for *L. trochiformis*, ATP6, COI for *L. lesueurii*, ATP6, COII, 16S for *L. helicina*, and only 16S for *H. inflatus*. Additionally, for *L. trochiformis*, seven biomineralisation candidates and four non-coding targeted regions were recovered. The number of SNPs ranged between 1371 (*H. inflatus*) and 12,165 SNPs (*L. trochiformis*) based on a gentoyping rate of 80% and a minimum read depth 5x (Table 5). The maximum depth for SNPs ranged from ~150x in *H. inflatus*, *L. helicina* and *L. lesueurii* to ~375x in *L. trochiformis* (Fig. 3). With less stringent filtering, such as a 50% genotyping rate, the total number of SNPs obtained per species could be increased (Table 5).

**Fig. 2:**
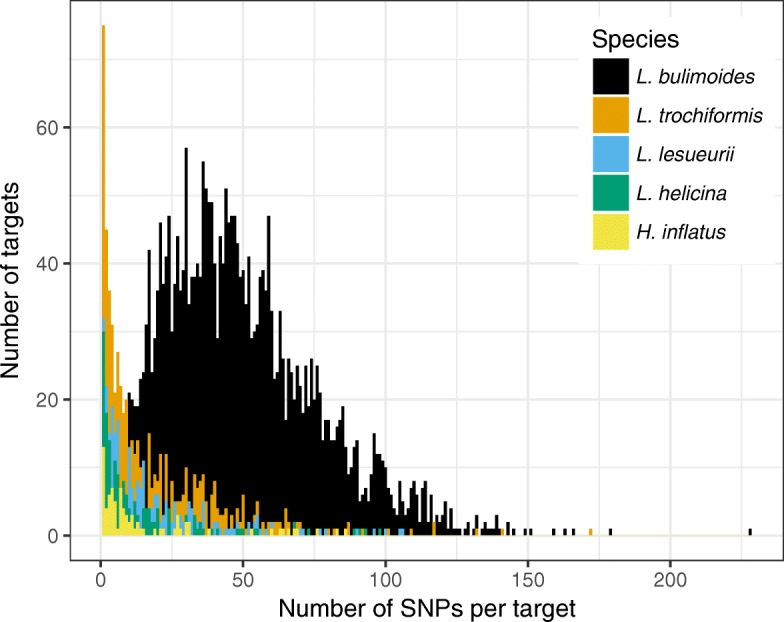
Number of single nucleotide polymorphisms (SNPs) per recovered target for the five pteropod species of the superfamily Limacinoidea (see legend), based on filtering settings of minimum presence in 80% of individuals with at least 5x read depth

Across the five species of Limacinoidea, we found an exponential decrease in the efficiency of the targeted re-sequencing congruent with the genetic distance from the focal species *L. bulimoides*. Only 62 targets were found in common across all five species, comprising 14 conserved pteropod orthologues, 47 coding regions, and a 700 bp portion of the 28S nuclear gene. Based on the differences in profiles of number of SNPs per target and total number of SNPs, the hybridisation worked differently between the focal and non-focal species. In *L. bulimoides*, the median number of SNPs per target was 45, whereas in the remaining four species, most of the targets had only one SNP and the median number of SNPs per target was much lower: 11 for *L. trochiformis*, 10 for *L. lesueurii*, six for *L. helicina*, and seven for *H. inflatus*. The number of SNPs per target varied between one and more than 200 across the targets (Fig. 2). With an increase in genetic distance from *L. bulimoides,* the total number of SNPs obtained across the five shelled pteropod species decreased exponentially (Fig. 4). There was an initial 10-fold decrease in number of SNPs between *L. bulimoides* and *L. trochiformis* with a maximum likelihood (ML) distance of 0.07 nucleotide substitutions per base between them. The subsequent decrease in number of SNPs was smaller in *L. lesueurii* (ML distance from *L. bulimoides*, subsequently ML dist = 0.11), *L. helicina* (ML dist = 0.18) and *H. inflatus* (ML dist = 0.29).

## Discussion

### First draft genome for pteropods

To assess the genetic variability and degree of population connectivity in coiled-shell pteropods, we designed a set of target capture probes based on partial genomic and transcriptomic resources. As a first step, we de novo assembled a draft genome for *L. bulimoides*, the first for a planktonic gastropod. We obtained an assembly size of 2.9 Gbp but the prediction of genome size together with the prediction of genome completeness suggest that only ~ 60% of the genome was sequenced. Therefore, we postulate that the genome size of *L. bulimoides* is indeed larger than the assembly size, and estimate it at 6–7 Gbp. In comparison, previously sequenced molluscan genomes have shown a wide variation in size across species, ranging from 412 Mbp in the giant owl limpet (*Lottia gigantea)* [47] to 2.7 Gbp in the Californian two-spot octopus (*Octopus bimaculoides)* [48]. The closest species to pteropods which has a sequenced genome is *Aplysia californica*, with a genome size of 927 Mbp (Genbank accession assembly: GCA_000002075.2) [36, 49]. Further, when considering marine gastropod genome size estimates in the Animal Genome Size Database [50], genome sizes range from 430 Mbp to 5.88 Gbp with an average size of 1.86 Gbp. Hence, it appears that *L. bulimoides* has a larger genome size than most other gastropods.

Despite moderate sequencing efforts, our genome is highly fragmented. Increasing the sequencing depth could result in some improvements, although other sequencing methods will be required to obtain a better genome. Roughly 350 million paired-end (PE) reads were used for the de novo assembly, but 50% of the assembly is still largely unresolved with fragments smaller than 893 bp. The absence of peaks in the k-mer distribution histogram and low mean coverage of the draft genome may indicate insufficient sequencing depth caused by a large total genome size, and/or high heterozygosity which complicates the assembly. In the 1.6 Gbp genome of another gastropod, the big-ear radix, *Radix auricularia*, approximately 70% of the content consisted of repeats [51]. As far as we know, high levels of repetitiveness within molluscan genomes are common [52], and also makes de novo assembly using only short reads challenging [53]. In order to overcome this challenge, genome sequencing projects should combine both short and long reads to resolve repetitive regions that span across short reads [54, 55]. Single molecule real time (SMRT) sequencing techniques which produce long reads recommend substantial DNA input, although some recent developments in library preparation techniques have lowered the required amount of DNA [56]. These SMRT techniques also tend to be high in cost, which may be a limiting factor when choosing between sequencing methods. Constant new developments in sequencing-related technologies may soon bring the tools needed to achieve proper genome assembly even for small-sized organisms with large genomes. Potential methods to improve current shotgun assemblies include 10x Genomics linked-reads [57] that uses microfluidics to leverage barcoded subpopulations of genomic DNA or Hi-C [58], which allow sequences in close physical proximity to be identified as linkage groups and enable less fragmented assemblies.

### Target capture probes for *Limacina bulimoides*

Our results show that generating a draft genome and transcriptome to serve as a reference in the design of target capture probes is a promising and cost-effective approach to allow population genomics studies in non-model species of small sizes. Despite the relatively low N50 of the assembled genome, we were able to map 79.8% of the transcript sequences onto it. The combined use of the transcriptome and fragmented genome allowed us to identify the expressed genomic regions reliably and include intronic regions, which may have contributed to the probe hybridisation success [59]. In addition, the draft genome was useful in obtaining single-copy regions. This allowed us to filter out multi-copy regions at the probe design step, and hence reducing the number of non-target matches during the capture procedure.

**Fig. 3:**
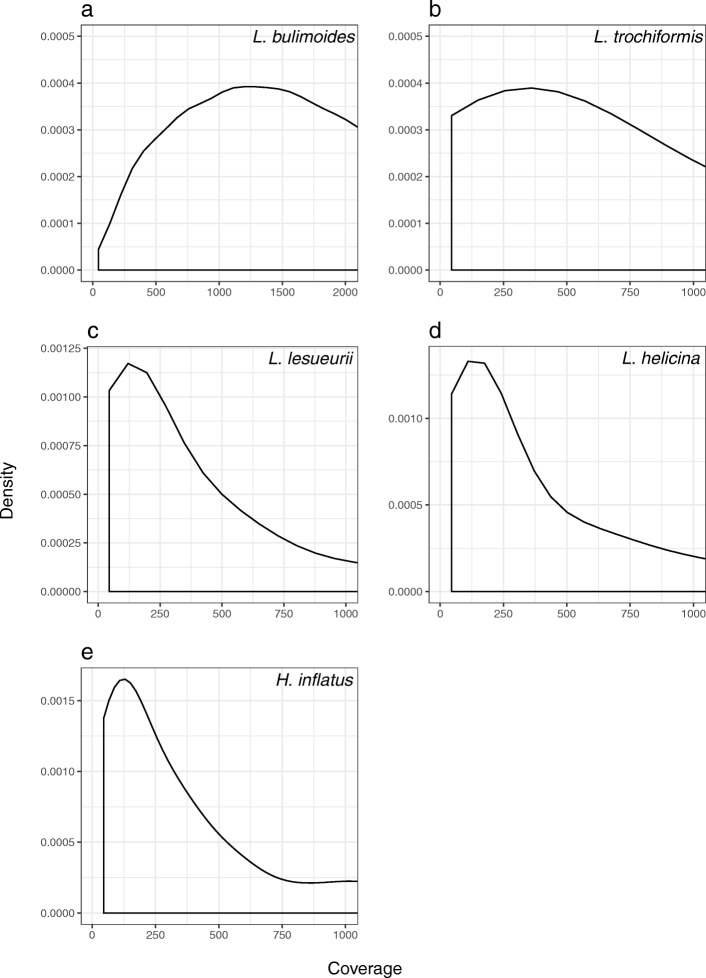
Density of single nucleotide polymorphisms (SNPs, present in 80% of individuals) plotted against coverage for each of the five pteropod species (**a**: *Limacina bulimoides*, **b**: *L. trochiformis*, **c**: *L. lesueurii*, **d**: *L. helicina*, and **e**: *Heliconoides inflatus*). The plots were truncated at coverage = 2000x for *L. bulimoides* and coverage = 1000x for the other four species. Note that minimum coverage is 45x due to filtering settings of a minimum 5x depth for 9 individuals

The target capture was highly successful in the focal species *L. bulimoides*, with more than 130,000 SNPs recovered across nine individuals (Fig. 3). Coverage of reads across the recovered targets was somewhat variable (Additional file 1: Figure S2A in Appendix S2), although the SNPs were obtained from the large proportion of sufficiently well-covered targets (>15x, Table 4; Additional file 1: Table S1) and thus, can provide reliable genomic information for downstream analyses, such as delimiting population structure. The high number of SNPs may be indicative of high levels of genetic variation, congruent with predictions for marine zooplankton with large population sizes [12]. The number of SNPs recovered (Table 4) and percentage of properly paired reads mapping uniquely to the targets (Table 3) are comparable to the results from a similar protocol on copepods [26].

Targets corresponding to candidate biomineralisation genes and mitochondrial genes were less successfully recovered compared to conserved pteropod orthologues and other coding sequences (Table 4). This could be because biomineralisation-related gene families in molluscs are known to evolve rapidly, with modular proteins composed of repetitive, low complexity domains that are more likely to accumulate mutations due to unequal cross-over and replication slippage [60, 61]. Surprisingly, only the COI gene was recovered out of the 10 mitochondrial genes included in the set of probes. This is despite the theoretically higher per cell copy number of mitochondrial than nuclear genomes [62] and thus a higher expected coverage for mitochondrial targets compared to nuclear targets. High levels of mitochondrial polymorphism among individuals of *L. bulimoides* could have further complicated the capture, resulting in low capture success of mitochondrial targets. Hyperdiversity in mitochondrial genes, with more than 5% nucleotide diversity in synonymous sites has been reported for several animal clades, including gastropods [63, 64] and chaetognaths [65]. Only 13 of the 41 non-coding targeted regions were recovered, which may indicate that these regions were also too divergent to be captured by the probes.

### Cross-species relevance of target capture probes

**Fig. 4:**
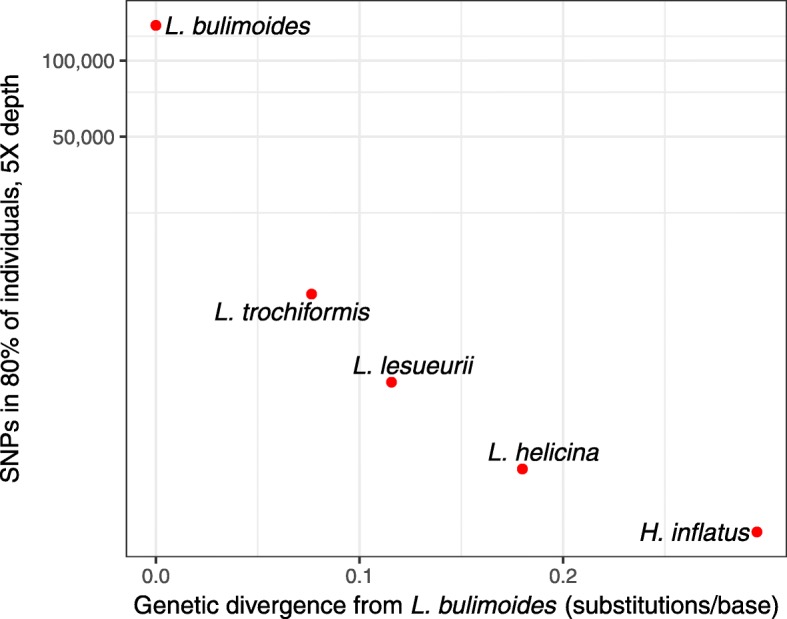
Log-scaled number of SNPs against genetic divergence from the focal species *Limacina bulimoides* shows that there is a sharp reduction in the SNPs recovered with genetic distance

The success of targeted re-sequencing of the four related pteropod species (*L. trochiformis*, *L. lesueuri*, *L. helicina* and *Heliconoides inflatus*) decreased exponentially with increasing genetic distance from the focal species *L. bulimoides*. Even within the same genus, divergence was sufficiently high to show an abrupt decrease in coverage (Fig. 3). The number of targets whose reads accounted for 50% of all reads for each species was low (Additional file 1: Figure S2B-E in Appendix S2), indicating that representation across the targets could be highly uneven. The number of SNPs recovered also decreased rapidly with genetic distance (Fig. 4), leading to less informative sites across the genome that can be used in downstream analyses for these non-focal species. While direct comparisons are not possible due to differences in the probe design protocol and measurements used, we also see a decreasing trend in success of target capture applied with increasing levels of genetic divergence in other studies e.g. [66, 67]. Genetic divergence of 4–10% from the focal species resulted in an abrupt decline in coverage e.g. [62, 68]. Another possible reason for the decrease in capture success is different genome sizes across the species. While we used the same amount of DNA per individual in a capture reaction, pooling different species of unknown genome sizes into the same capture reaction may have resulted in different genome copy numbers sequenced per species. Our results may thus be attributed to high levels of polymorphism and/or possible differences in genome size, both leading to ascertainment bias [69].

The targets that hybridised successfully and were sequenced across species were conserved genes with low levels of genetic variation. This probably indicates that high levels of genetic diversity and divergence from the focal species resulted in the targeted regions not being able to hybridise to the probes. Indeed, from the four non-focal pteropod species, most of the recovered targets had low diversity, containing only a single SNP (Fig. 2). As a general rule, slowly evolving genomic regions are more likely to hybridise successfully to the probes [33, 70]. This may vary across targeted regions, as a mismatch tolerance of 40% between the baits and targeted region can still result in successful enrichment in specific cases [71]. While it is possible to design probes to be relevant across broader phylogenetic scales, by including conserved orthologues across the various target species e.g. [72, 73], these probes are unlikely to be suitable to study population structure and estimate levels of gene flow in the focal species. Nonetheless, the low diversity targets that were recovered can be useful in resolving relationships at a deeper phylogenetic scale.

## Conclusion

We show that using a combination of a draft genome and transcriptome is an efficient way to develop a database for capture probes design in species without prior genomic resources. These probes can be useful for analyses in closely related species, though cross-species hybridisation was limited to conserved targets and capture success decreased exponentially with increasing genetic distance from the focal species. Since the target capture approach can be successfully applied with low DNA input and even with poor quality or degraded DNA, this technique opens the door to population genomics of zooplankton, from recent as well as historical collections.

With more than 130,000 SNPs recovered in *L. bulimoides* and > 10,000 SNPs in *L. trochiformis*, our set of probes is suitable for genome-wide genotyping in these two globally distributed pteropod species. The high and consistent coverage across targeted genomic regions increases the range of analyses that can be applied to these organisms, such as identifying dispersal barriers, inferring ancestry and demographic history, and detecting signatures of selection across the genome. The statistical strength from analysing many genomic loci overcomes the limitation of an incomplete sampling of the metapopulation [74] and increases the capacity to detect even subtle patterns in population structure. This is especially relevant in widespread marine zooplankton where there is likely to be cryptic diversity and undiscovered species [12, 20], which is essential information for species that are proposed as indicators of ocean change.

## Methods

### Draft genome sequencing and assembly

A single adult *L. bulimoides* (1.27 mm total shell length) was used to generate a draft genome (NCBI: SWLX00000000). This individual was collected from the southern Atlantic subtropical gyre (25°44′S, 25°0′W) during the Atlantic Meridional Transect (AMT) cruise 22 in November 2012 (Additional file 1: Appendix S3 and Figure S3) and directly preserved in 95% ethanol at − 20 °C. Back in the lab, 147.2 ng of genomic DNA was extracted from the whole specimen using the E.Z.N.A. Insect DNA Kit (Omega Bio-Tek) with modifications to the manufacturer’s protocol regarding reagents volumes and centrifuge times (Additional file 1: Appendix S3). The extracted DNA was randomly fragmented via sonication on a S220 Focused-ultrasonicator (Covaris) targeting a peak length of approximately 350 bp. A genomic DNA library was prepared using the NEXTflex Rapid Pre-Capture Combo Kit (Bioo Scientific) following the manufacturer’s protocol. Subsequently, the library was sequenced in two runs of NextSeq500 (Illumina) using mid-output v2 chips producing 150 bp PE reads.

The resulting forward and reverse sequencing reads were concatenated in two separate files and quality-checked using FastQC version 0.11.4 [75]. Duplicated reads were removed using FastUniq version 0.11.5 [76]. The remaining reads were then assembled by the MaSuRCA genome assembler version 3.2.1 [41] using a k-mer length of 105 as this produced the least fragmented assembly compared to other assemblers (Platanus, SOAPdenovo2). Further contig extension and scaffolding were carried out by running SSPACE-Basic version 2 [77] requiring a minimum of three linkers and a minimum overlap of 12 bp to merge adjacent contigs [77]. The total genome size was roughly estimated using MaSuRCA (as a by-product of calculating optimal assembly parameters), based on the size of the hash table containing all error corrected reads. A second estimate of the genome size was made by searching for k-mer peaks in sequencing reads using JELLYFISH version 1.1.11 [40] with various k-mer lengths between 15 and 101. To assess the completeness of the generated draft genome, the in-built BUSCO metazoan dataset containing 978 near-universal orthologues of 65 species was used to search for key orthologous genes with BUSCO version 3.0.1 [42]. BUSCO made use of AUGUSTUS version 3.3 [78] with the self-training mode utilised to predict gene models. Assembly quality was assessed with QUAST [79].

### Target capture probes design

We designed the target capture probe set by using the draft genome and transcriptome as a reference, following the workflow recommended by Choquet et al. [26]. Firstly, we aimed to select only single-copy coding DNA sequences (CDS) in order to achieve a high specificity of the target capture probes and to reduce false-positive SNPs from multi-copy genes. We used the previously generated transcriptome of *L. bulimoides* [43] and mapped the transcript sequences of *L. bulimoides* against themselves using the splice-aware mapper GMAP version 2017-05-03 [44] with a k-mer length of 15 bp and no splicing allowed. Only unique transcripts with one mapping path were selected as potential target sequences. We then mapped these selected transcript sequences (with splicing allowed) directly to the contigs of the genomic assembly to identify expressed regions and their respective exon-intron boundaries. We selected only the subset of genomic sequences that mapped to unique transcripts with minimum pairwise identity scores of 90%. Using this approach, we selected 2169 coding target sequences. Additionally, 643 transcripts that mapped to unique contigs in the draft genome were selected from a set of conserved orthologues from a phylogenomic analysis of pteropods [43] to give a set of 2812 single copy coding nuclear targets. Of the 63 transcripts that showed homology to biomineralisation proteins [45, 46], we included 35 of these candidate biomineralisation genes in the final probe set as they could be mapped to contigs in the draft genome (Additional file 2).

Secondly, sequences of mitochondrial genes, 28S and non-coding targets were added to the baits design. A fragment of the COI gene (NCBI: MK642914), obtained by sanger sequencing as in [37] was added. The other nine targets (COII, COIII, ATP6, ND2, ND3, ND6, CYB, 12S, 16S) were identified from the draft genome assembly as described hereafter. We identified a 9039 bp contig from the fragmented assembly as a partially assembled mitochondrial genome using BLAST+ version 2.6.0 [80] and comparing the mitochondrial genes of three related mollusc species (NCBI Bioprojects: PRJNA10682, PRJNA11892, PRJNA12057) to the draft genome. Gene annotation was then carried out on this contig using the MITOS webserver [81] with the invertebrate genetic code and the parameters ‘cut-off’, ‘fragment quality factor’ and ‘start/stop range’ set to 30, 12 and 10, respectively. From this, we identified the seven protein-coding genes and the two rRNA genes as separate target sequences which we added to the probe design. Finally, we added the commonly-used nuclear 28S Sanger-sequenced fragment (NCBI: MK635470) and randomly chose 41 unique non-coding genomic regions. The final design comprised of 2899 target sequences with a total size of 1,866,005 bp. Probe manufacturing was performed by Arbor Biosciences (MI, USA) using myBaits custom biotinylated probes of 82-mer with 2x tiling density (Additional file 3).

### Targeted sequencing of five pteropod species

We selected five shelled pteropod species from the genera *Limacina* and *Heliconoides* (superfamily Limacinoidea), including the focal species *L. bulimoides*, to evaluate the efficiency of the target capture probes on species of varying genetic relatedness. For each species, we aimed to test the capture efficiency across three sampling locations with three individuals per location (Table 6). Specimens from each species (*L. bulimoides*, *L. trochiformis*, *L. lesueurii*, *L. helicina*, *H. inflatus*) were collected across various sites during the AMT22 and AMT24 cruises in the Atlantic and from two sites in the Pacific Ocean (Table 6 and Additional file 1: Table S2). DNA was extracted from each individual separately using either E.Z.N.A. insect or mollusc kit (Omega Bio-Tek) with modifications to the protocol (Additional file 1: Appendix S3). The DNA was then sheared by sonication, using a Covaris S220 ultrasonicator with the peak length set to 300 bp. This fragmented DNA was used to prepare individual libraries indexed using the NEXTflex Rapid Pre-Capture Combo Kit (Bioo Scientific). Libraries were subsequently pooled into equimolar concentrations for the capture reaction using the myBaits Custom Target Capture kit (Arbor Biosciences). Hybridisation was carried out using the myBaits protocol with the following modifications. Twenty-seven libraries of *L. bulimoides* were pooled together for one capture reaction, of which nine individuals were analysed in this study. The other four species were pooled in groups of 22–23 specimens per capture. We extended the hybridisation time to 3 days and performed the whole protocol twice using 4 μL and 1.5 μL of probe mix, respectively (Additional file 1: Appendix S3). The captured library of the species *L. bulimoides* was sequenced on the NextSeq500 (Illumina) using a high-output v2 chip producing 150 bp PE reads. The captured libraries of the other species were sequenced together on the same NextSeq500 mid-output v2 chip.

**Table 6:** Collection details of specimens from five shelled pteropod species: *Limacina bulimoides*, *L. trochiformis*, *L. lesueurii*, *L. helicina* and *Heliconoides inflatus*. Three individuals per site were included from localities in the Atlantic and Pacific Oceans. Latitude and longitude are presented in the decimal system, with positive values indicating North and East and negative values, South and West, respectively

Species	Location	Latitude	Longitude	n	Collection Date
*L. bulimoides*	South Atlantic	−18.32	−25.08	3	18/10/2014
*L. bulimoides*	South Atlantic	− 24.45	−25.05	3	21/10/2014
*L. bulimoides*	South Atlantic	−27.77	−25.02	3	22/10/2014
*L. trochiformis*	South Atlantic	−14.67	−25.07	3	17/10/2014
*L. trochiformis*	South Atlantic	−18.32	−25.08	3	18/10/2014
*L. trochiformis*	North Pacific	22.65	− 157.69	3	03/07/2017
*L. lesueurii*	North Atlantic	20.40	−38.61	3	24/10/2012
*L. lesueurii*	South Atlantic	−15.30	−25.07	3	05/11/2012
*L. lesueurii*	South Atlantic	−24.13	−25.00	3	09/11/2012
*L. helicina*	South Atlantic	−40.12	−30.92	3	26/10/2014
*L. helicina*	South Atlantic	−41.48	−33.87	3	27/10/2014
*L. helicina*	North Pacific	48.36	−126.31	3	06/03/2016
*H. inflatus*	North Atlantic	25.48	−39.00	3	22/10/2012
*H. inflatus*	South Atlantic	−8.08	−25.04	3	03/11/2012
*H. inflatus*	South Atlantic	−38.08	−39.31	3	16/11/2012

### Assessment of target capture probes efficiency

The following pipeline of bioinformatic analyses was largely adapted from Choquet et al. [26]. Raw sequencing reads were de-multiplexed and mapped using BWA version 0.7.12 [82] with default settings to targets concatenated with the perl script concatFasta.pl [83]. The resulting BAM files were then cleaned and sorted using SAMtools version 1.4.1 [84] to retain only the reads paired and uniquely mapped in proper pairs. With Picard version 2.18.5 [85], duplicates were marked and removed. Coverage of targeted regions was assessed with the GATK version 3.8 [86] DepthOfCoverage tool. Next, SNP calling was performed using GATK version 3.8 with GNU Parallel [87] following the recommended Variant Discovery pipeline [88, 89] as a first trial for SNP calling in pteropods. Variants were called per individual using HaplotypeCaller with emitRefConfidence output, and the resulting gVCF files were combined according to their species with CombineGVCFs. The combined gVCF files for each species, with nine individuals each, were then genotyped in GenotypeGVCFs. SNPs were extracted from the raw variants with SelectVariants (−SelectType SNP). Given the lack of a calibration set of SNPs, the hard filters were first evaluated by plotting the density of annotation values and checking them against the planned filtering parameters. The SNPs were then hard-filtered with VariantFiltration using QualByDepth (QD) < 2.0, FisherStrand (FS) > 60.0, RMSMappingQuality < 5.0, MQRankSumTest (MQRankSum) < − 5.0, ReadPositionRankSum (ReadPosRankSum) < − 5.0 to retain reliable SNPs. The processed SNPs were further filtered using VCFtools version 0.1.13 [90] to keep those with a minimum coverage of 5x and represented in at least 80% of the individuals.

In order to investigate the relative effect of the different SNP filters, other less conservative VCFtools filtering settings such as a reduced genotyping rate of 50% or reduced depth requirement of 2x were used, and the relative increase in number of SNPs recovered for each species was recorded. For each species, the resulting VCF files were then annotated with the names and coordinates of the original targets using retabvcf.pl [83]. The targets represented in each species and the number of SNPs per target were then extracted from the annotated VCF files (Additional file 1: Appendix S4).

To assess the applicability of probes designed from *L. bulimoides* and other related pteropod species, the relationship between sequence divergence and number of SNPs recovered was investigated. The genetic divergence between *L. bulimoides* and each of the four other species was calculated from the branch lengths of a maximum likelihood (ML) phylogeny of pteropods based on transcriptome data [43]. The number of SNPs recovered per species using the most conservative filtering settings (80% genotyping rate and 5x depth) was plotted against sequence divergence from *L. bulimoides* in R [91].

## Supplementary information

**Additional file 1:** Supporting information containing Appendices S1-5 and Tables S1-2.

**Additional file 2:** Transcripts of *L. bulimoides* with homology to biomineralisation proteins.

**Additional file 3:** 82-mer probe sequences for *L. bulimoides*.

## Data Availability

The genomic assembly (NCBI accession: SWLX00000000, BioSample ID: SAMN11131519), and raw sequencing data of the target capture are available in NCBI Genbank, under BioProject PRJNA527191. The transcriptome is available in NCBI Genbank under the NCBI accession SRR10527256 (BioSample ID: SAMN13352221, BioProject: PRJNA591100). The list of *L. bulimoides* contigs with homology to biomineralisation proteins and set of 82-mer probes developed for *L. bulimoides* are included as Additional file 2 and Additional file 3. The additional information supporting the conclusions of this article are included as appendices within the Additional file 1.

## Abbreviations

AMT: Atlantic Meridional Transect
CDS: Coding DNA Sequence
COI: Cytochrome Oxidase subunit I
ML: Maximum Likelihood
NGS: Next Generation Sequencing
PE: Paired End
SMRT: Single Molecule Real Time
SNP: Single Nucleotide Polymorphism
