# Supplementary material for: Novel genomic resources for shelled pteropods: a draft genome and target capture probes for Limacina bulimoides, tested for cross-species relevance
Source: BMC Genomics. 2020 Jan 3;21:11. doi: 10.1186/s12864-019-6372-z (PMC6942316; doi:10.1186/s12864-019-6372-z)
Supplement: Supplementary file 1 — Additional file 1: Supporting information containing Appendices S1-5 and Tables S1-2. [file 12864_2019_6372_MOESM1_ESM.docx]

**Supporting information for:**

**Novel genomic resources for shelled pteropods: a draft genome and target capture probes for *Limacina bulimoides*, tested for cross-species relevance**

L.Q. CHOO^1,3^*, T.M.P. BAL^2^*, M. CHOQUET^2^, I. SMOLINA^2^, P. RAMOS-SILVA^1^, F. MARLÉTAZ^4^, M. KOPP^2^, G. HOARAU^2^, K.T.C.A. PEIJNENBURG^1,3^

*^1^Marine biodiversity, Naturalis Biodiversity Center, Leiden, 2300 RA, the Netherlands, ^2^Faculty of Biosciences and Aquaculture, Nord University, 8049 Bodø, Norway, ^3^Institute for Biodiversity and Ecosystem Dynamics (IBED), University of Amsterdam, Amsterdam, 1090 GE, the Netherlands,* ^4^*Molecular Genetics Unit, Okinawa Institute of Science and Technology, Onna-son, 904-0495, Japan.*

*Shared first authorship

Correspondence to L.Q. Choo at: [leqin.choo@naturalis.nl](mailto:leqin.choo@naturalis.nl) and K.T.C.A. Peijnenburg at: [K.T.C.A.Peijnenburg@uva.nl](mailto:K.T.C.A.Peijnenburg@uva.nl).

***Appendix S1*** *Draft genome statistics*

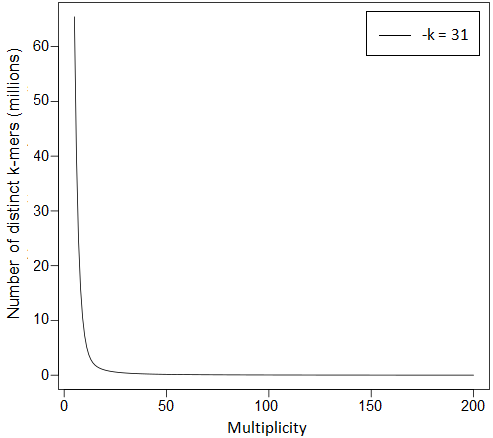


**Fig S1.** Histogram of k-mer frequency distribution in the assembled *L. bulimoides* draft genome for k = 31. The x-axis represents the number of times a k-mer occurred and the y-axis represents the number of distinct k-mers for the given multiplicity.

***Appendix S2*** *Density of coverage per target for each species*


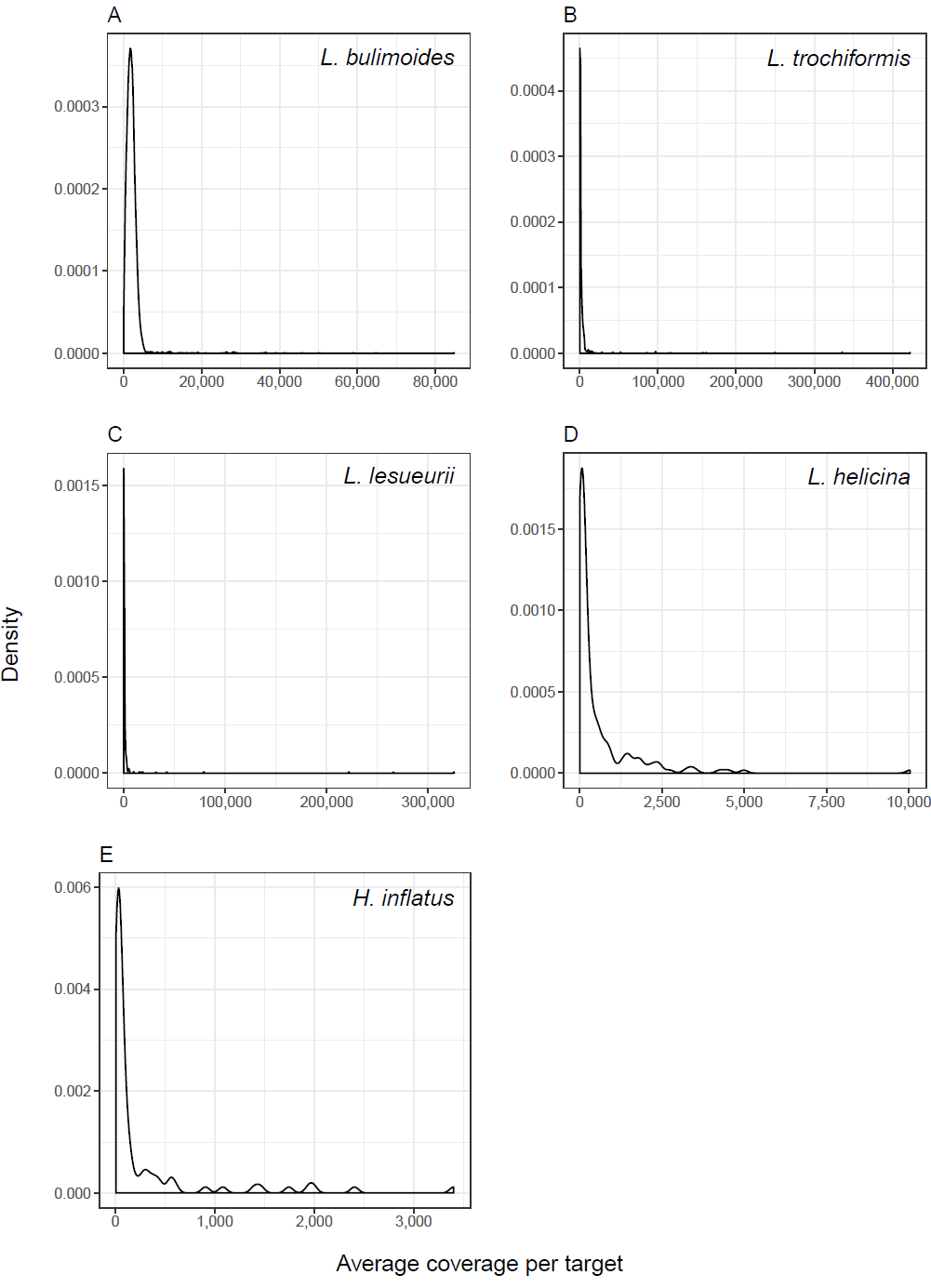


**Fig S2** Density plot of coverage for each target, averaged across nine individuals, for each of the five shelled pteropod species (*L. bulimoides*, *L. trochiformis*, *L. lesueurii*, *L. trochiformis*, *L. helicina* and *H. inflatus*).

***Appendix S3*** *Specimen collection and molecular analyses*

**Draft genome**

The sequenced *L. bulimoides* individual, Lbul_AMT22_57_08 was collected during the Atlantic Meridional Transect 22 (AMT22) cruise in November 2012 in the southern gyre of the Atlantic Ocean (25°44'S, 25°0'W). The specimen was photographed in a standardised orientation using a Zeiss V20 stacking microscope (Fig. S1).


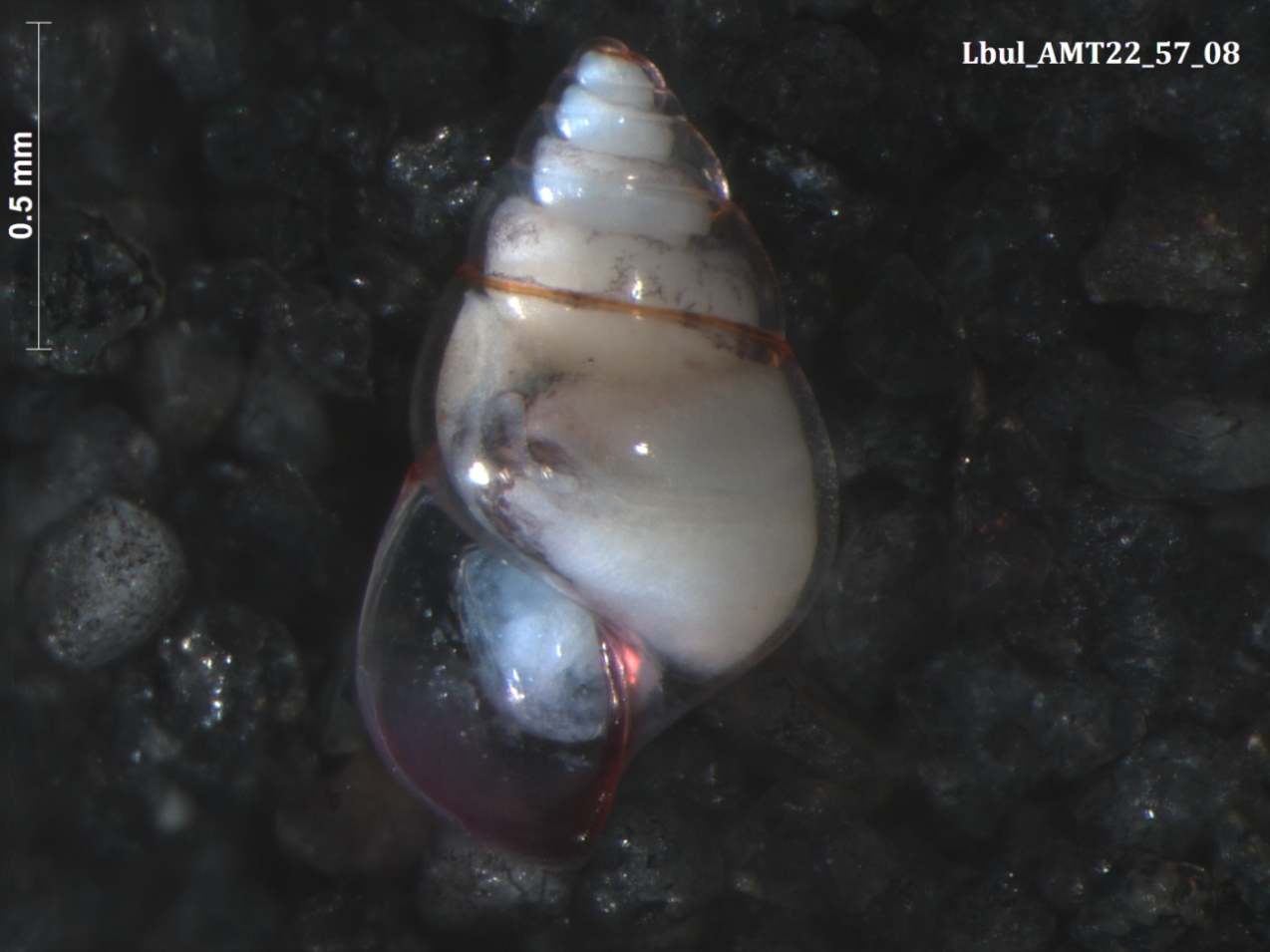


**Fig S3.** Stacking microscopy photograph of the sequenced *Limacina bulimoides* specimen.

**Modified DNA extraction protocol with E.Z.N.A Insect Kit (Omega Bio-tek) for extracting DNA from shelled pteropods**

*1.* Soak the complete individual in nuclease-free water for 30 minutes.

*2.* Transfer individual into 2 ml screw cap grinding tube containing:

- Bashing beads
- 400 μl CTL buffer
- 28.5 μl Proteinase K solution

*3.* Grind individual for 20 seconds at a frequency of 30 per second.

*4.* Incubate sample at 60 °C for 20 minutes.

*5.* Repeat step *3* and incubate sample at 60°C for 20 minutes.

*6.* Add 400 μl chloroform:isoamyl alcohol, vortex for 10 seconds.

*7.* Centrifuge at 13,500 g for 18 minutes at room temperature.

*8*. Prepare the HiBind DNA Mini Column:

- Assemble to collection tube
- Add 100 μl 3 M NaOH (equilibration buffer) on filter
- Incubate for 4 minutes and centrifuge at 17,000 g for 20 seconds.

*9.* Transfer upper aqueous phase (~320 μl) from step 7 to a new 1.5 ml eppendorf tube. Avoid the white milky surface.

*10*. Add one volume CBL buffer.

*11.* Add one volume 100% EtOH and vortex for 10 seconds.

*12*. Transfer 750 μl of the mixture to the equilibrated HiBind DNA Mini Column and centrifuge at 10,000 g for 1 minute. Discard the flow-through.

*13.* Repeat step 12 until all of the mixture has been used.

*14*. Warm-up the Elution Buffer at 70°C.

*15.* Add 500 μl HBC buffer and centrifuge at 10,000 g for 30 seconds. Discard the flow-through.

*16.* Add 500 μl DNA Wash Buffer and centrifuge at 10,000 g for 1 minute. Discard the flow-through.

*17*. Repeat step 15.

*18.* Re-insert HiBind DNA Mini Column into new collection tube, centrifuge at 15,000 g for 2 minutes.

*19.* Re-insert HiBind DNA minicolumn into new eppendorf tube.

*20.* Add 40 μl of pre-heated Elution Buffer directly to the center of the Mini Column membrane without touching it.

*21.* Wait for 2 minutes and then centrifuge at 10,000 g for 1 minute.

*22.* Repeat steps 20 and 21 for a final elution volume of 80 μl.

**Target capture protocol**

All DNA libraries were fragmented to an average size of 300 bp by sonication and were prepared using the NEXTflex™ Rapid Pre-Capture Combo Kit (Bioo Scientific, Austin, TX, USA), including a step of single adapter indexing of each library. Libraries were cleaned-up, amplified separately for 8 and pooled in the following way: the nine *L. bulimoides* were part of a pooled capture that included 27 specimens of *L. bulimoides* in total. The four other species were amplified separately for another 8 cycles to increase the amount of DNA, and then combined into two pools that contained all four species with a total of 22-23 specimens per pool.

We increased the efficiency of the hybridisation and aimed to maximise the number of on-target captured sequences by doing the capture reaction twice, splitting the total amount of baits required for one reaction in two. The first round of hybridisation was performed using 4 μL of baits for each reaction. The reaction was performed over three days at a temperature of 60°C in order to maximise the specificity. Capture was performed consecutively using DYNAbeads MyOne Streptavidin C1 beads (Invitrogen) to bind the hybridised targets during 30 min at 65°C. The captured DNA was amplified by PCR for 8 cycles using KAPA HiFi HotStart ReadyMix (Kapa Biosystems). A second round of hybridisation was conducted using 1.5 μL of baits for each of the two pools, followed by a second capture and 6 more cycles of post-capture PCR. Finally, the two mixed-species pools were mixed together in equal proportions and sequenced on a NextSeq 550 (Illumina) with a 2x150 bp mid-output kit v.2. The *L. bulimoides* pool was mixed equally with two other *L. bulimoides* pools containing 27 individuals and sequenced on a NextSeq 550 (Illumina) with a 2x150 bp high-output kit v.2.

***Appendix S4*** *Commands used for SNP calling from fastq files*

For manipulating targets fasta file:

Used concatFasta.pl <https://github.com/z0on/2bRAD_denovo/blob/master/concatFasta.pl> (concatenate fasta file into user-specified number of “chromosomes”) and retabvcf.pl from <https://github.com/z0on/2bRAD_denovo/blob/master/retabvcf.pl> (re-annotate .vcf file) to increase speed and reduce memory requirements. This is done before mapping reads to the targets.

1. Use mapping.sh on demulitplexed, raw fastq.gz files.

Contents of mapping.sh:

##Mapping (input: *_R1.fastq.gz, *_R2.fastq.gz, <draft_genome_concatenated>.fasta)

## collect file names and place in file called names

ls *_R1.fastq.gz >names
sed -e s/_R1.fastq.gz//g -i names

## use parallel to run 24 threads of bwa mem, estimate two threads per specimen, for computer with 48 threads, producing aligned bam file *_aln.bam per specimen

cat names | parallel --verbose -j 24 "
bwa mem -M Genomic_targets_Limacina_bulimoides_cc.fasta {}_R1.fastq.gz {}_R2.fastq.gz | samtools view -Sbh - -o {}_aln.bam"

##cleaning initial bam files

cat names | parallel --verbose -j 10 "
samtools view {}_aln.bam | fgrep XA | cut -f 1 > bad_names_{}.txt
samtools view -h {}_aln.bam | fgrep -vf bad_names_{}.txt | samtools view -Sb - > {}_aln2.bam
samtools view {}_aln2.bam | fgrep SA | cut -f 1 > bad_names_{}.txt
samtools view -h {}_aln2.bam | fgrep -vf bad_names_{}.txt | samtools view -Sb - > {}_aln3.bam
samtools view -b -F 3332 -f 3 {}_aln3.bam > {}_aln3_cleaned.bam"

##sort reads (3min)
chmod 755 *_aln3_cleaned.bam
cat names | parallel --verbose -j 10 "
samtools sort {}_aln3_cleaned.bam -o {}_sorted.bam"

##mark duplicates

cat names | parallel --verbose -j 10 "
java -jar picard.jar MarkDuplicates I={}_sorted.bam O={}_dedup.bam M={}_dedup_metricsfile ASSUME_SORT_ORDER=coordinate VALIDATION_STRINGENCY=SILENT REMOVE_DUPLICATES=true &>Log_{}_dedup.txt"

##Add or replace read groups, most important is RGSM which allows GATK to call genotypes by individuals
cat names | parallel --verbose -j 15 "
java -jar picard.jar AddOrReplaceReadGroups I={}_dedup.bam O={}_dedup_RG.bam SORT_ORDER=coordinate RGLB=lib1 RGPL=illumina RGPU=unit54 RGSM={}"

##index .bam file

for file in *_RG.bam; do
samtools index $file "${file}.bai";
done

#end of mapping.sh

1. The steps below follow Best Practices for GATK3.8., for genotyping SNPs from bam files.

##HaplotypeCaller, use gnu-parallel to use multiple cores

cat names | parallel --verbose -j 20 "java -jar GenomeAnalysisTK.jar \
-R Genomic_targets_Limacina_bulimoides_cc.fasta \
-T HaplotypeCaller \
-I {}_dedup_RG.bam \
--emitRefConfidence GVCF \
-o $src/gvcf/{}.g.vcf"

##CombineGVCFs- files are combined per population at this step, so parallel is no longer needed.

java -jar GenomeAnalysisTK.jar \
-R ../Genomic_targets_Limacina_bulimoides_cc.fasta \
-T CombineGVCFs \
--variant Ltro.list \
-o Ltro.g.vcf

##GenotypeGVCFs

java -jar GenomeAnalysisTK.jar
-R ../Genomic_targets_Limacina_bulimoides_cc.fasta \
-T GenotypeGVCFs \
--variant Ltro.g.vcf \
--max_alternate_alleles 18 \ #depends on organism (diploid) and number of individuals (n=9)
-o ../combined/Ltro_raw_snps.vcf

##SelectVariants

for file in *.vcf; do
java -jar GenomeAnalysisTK.jar \
 -T SelectVariants \
 -R ../Genomic_targets_Limacina_bulimoides_cc.fasta \
 -V $file\
 -selectType SNP \
 -o ${file%_variants*}_snps.vcf;
done

##VariantFiltration

for file in *_raw_snps.vcf; do
java -jar GenomeAnalysisTK.jar \
-T VariantFiltration \
-R ../Genomic_targets_Limacina_bulimoides_cc.fasta \
-V $file \
--filterExpression "QD < 2.0 || FS > 60.0 || MQ < 50.0 || MQRankSum < -5.0 || ReadPosRankSum < -5.0" \
--filterName "choquet2018" \
-o ${file%raw*}filtered_snps.vcf &> ${file%raw*}filtered_snps.log ;
done

1. The steps below use vcftools to filter the snps within the vcf file and retabvcf.pl to rename coordinates of concatenated contigs with the names of the targets.

##Filter SNPs

for file in *filtered_snps.vcf; do
vcftools --vcf $file --max-missing 0.8 --min-meanDP 5.0 --recode --recode-INFO-all --out ${file%filtered*}cleaned_80_minDP5;
done

##Rename “chromosomes” in vcf file with name of the targets

for file in *_cleaned_80_minDP5.recode.vcf; do
~/2bRAD_denovo/retabvcf.pl vcf=$file tab=../Genomic_targets_Limacina_bulimoides_cc.tab >retab_$file;
done

##To count number of targets present in the vcf file

for file in retab_*; do
awk '/#CHROM/,0' $file |cut -f1 |uniq -c > ${file%cleaned*}target_uniqc.list;
done

***Appendix S5*** *Number of SNPs per target (individual plots)*


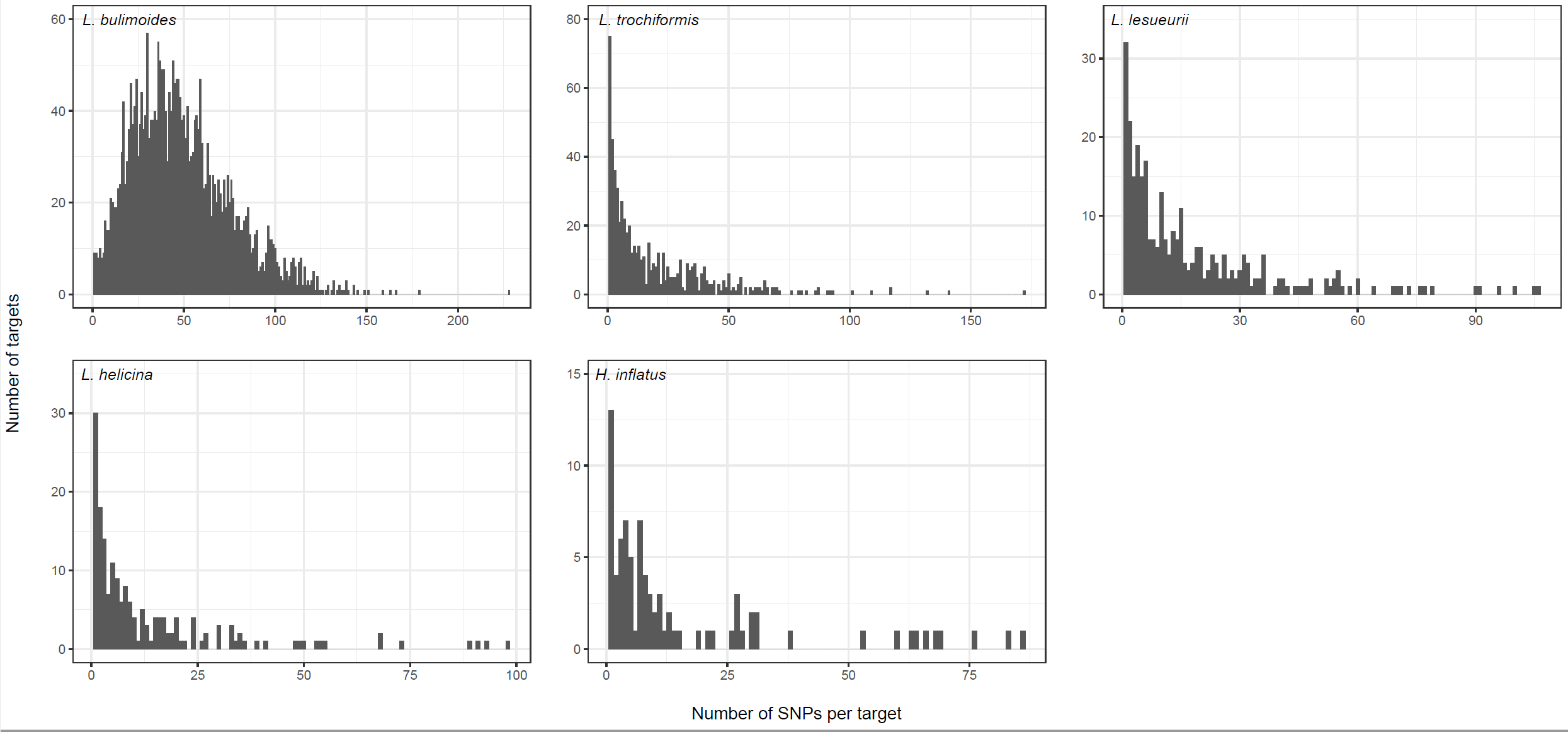


**Fig S4.** Number of SNPs per recovered target for each of the five shelled pteropod species (*Limacina bulimoides*, *L. trochiformis*, *L. lesueurii*, *L. helicina* and *Heliconoides inflatus*), based on filtering settings of minimum presence in 80% of individuals with at least 5x read depth.

## *Table S1 Target recovery with ≥15x coverage per species*

**Table S1.** Number of targets with a minimum coverage of 15x in at least one base, across the five shelled pteropod species *Limacina bulimoides*, *L. trochiformis*, *L. lesueurii*, *L. helicina* and *Heliconoides inflatus*. The number of targets with more than 90% or 50%, and less than 10% of bases with 15x coverage is also displayed for each species.

| Species | Total targets recovered at 15x depth or more | ≥90% bases recovered with 15x depth or more | ≥50% bases recovered with 15x depth or more | ≤10% bases recovered with 15x depth or more |
| --- | --- | --- | --- | --- |
| *L. bulimoides* | 2822 | 2446 | 2768 | 5 |
| *L. trochiformis* | 620 | 42 | 206 | 88 |
| *L. lesueurii* | 302 | 16 | 72 | 77 |
| *L. helicina* | 177 | 6 | 15 | 64 |
| *H. inflatus* | 83 | 5 | 13 | 44 |

***Table S2*** *Sampling localities and raw sequencing results per specimen.*

| Individual | RMNH number | NCBI | Latitude | Longitude | Raw reads | Final mapped reads | % HQ reads | % targets covered | Depth |
| --- | --- | --- | --- | --- | --- | --- | --- | --- | --- |
| Lbul_AMT24_20_03 | RMNH.MOL.341197 | SAMN11131474 | -18.32 | -25.08 | 9277276 | 4294018 | 46.29 | 97.52 | 311.44 |
| Lbul_AMT24_20_04 | RMNH.MOL.341198 | SAMN11131475 | -18.32 | -25.08 | 4997100 | 1044990 | 20.91 | 96.50 | 74.07 |
| Lbul_AMT24_20_09 | RMNH.MOL.341199 | SAMN11131476 | -18.32 | -25.08 | 9528311 | 3566992 | 37.44 | 97.40 | 251.49 |
| Lbul_AMT24_22_04 | RMNH.MOL.341200 | SAMN11131477 | -24.45 | -25.05 | 17168774 | 3718492 | 21.66 | 97.52 | 263.53 |
| Lbul_AMT24_22_06 | RMNH.MOL.341201 | SAMN11131478 | -24.45 | -25.05 | 6298310 | 1677720 | 26.64 | 96.94 | 113.11 |
| Lbul_AMT24_22_08 | RMNH.MOL.341202 | SAMN11131479 | -24.45 | -25.05 | 8394293 | 3357164 | 39.99 | 97.19 | 238.12 |
| Lbul_AMT24_23_06 | RMNH.MOL.341203 | SAMN11131480 | -27.77 | -25.02 | 10546514 | 2941122 | 27.89 | 97.77 | 204.06 |
| Lbul_AMT24_23_08 | RMNH.MOL.341204 | SAMN11131481 | -27.77 | -25.02 | 14110230 | 5391732 | 38.21 | 97.80 | 380.22 |
| Lbul_AMT24_23_09 | RMNH.MOL.3401205 | SAMN11131482 | -27.77 | -25.02 | 14440677 | 5786036 | 40.07 | 97.57 | 410.24 |
| Ltro_AMT24_19_01 | RMNH.MOL.340274 | SAMN11131501 | -14.67 | -25.07 | 19439184 | 2605782 | 13.40 | 22.75 | 693.79 |
| Ltro_AMT24_19_02 | RMNH.MOL.340275 | SAMN11131502 | -14.67 | -25.07 | 12770462 | 1741952 | 13.64 | 21.42 | 453.24 |
| Ltro_AMT24_19_03 | RMNH.MOL.340276 | SAMN11131503 | -14.67 | -25.07 | 13776593 | 1783082 | 12.94 | 20.23 | 474.98 |
| Ltro_AMT24_20_02 | RMNH.MOL.340277 | SAMN11131504 | -18.32 | -25.08 | 9152848 | 935860 | 10.22 | 19.40 | 238.24 |
| Ltro_AMT24_20_03 | RMNH.MOL.340278 | SAMN11131505 | -18.32 | -25.08 | 12020467 | 1979350 | 16.47 | 20.47 | 525.52 |
| Ltro_AMT24_20_05 | RMNH.MOL.340279 | SAMN11131506 | -18.32 | -25.08 | 11296879 | 1034722 | 9.16 | 17.13 | 283.11 |
| Ltro_KOK1703_03_01 | RMNH.MOL.340280 | SAMN11131507 | 22.65 | -157.69 | 16580239 | 1664588 | 10.04 | 22.03 | 408.59 |
| Ltro_KOK1703_03_02 | RMNH.MOL.340281 | SAMN11131508 | 22.65 | -157.69 | 22810256 | 2082476 | 9.13 | 19.53 | 571.05 |
| Ltro_KOK1703_03_03 | RMNH.MOL.341274 | SAMN11131509 | 22.65 | -157.69 | 21721894 | 2059844 | 9.48 | 19.91 | 564.29 |
| Lles_AMT22_25_01 | RMNH.MOL.341206 | SAMN11131483 | 20.40 | -38.61 | 5422102 | 894512 | 16.50 | 12.78 | 505.10 |
| Lles_AMT22_25_03 | RMNH.MOL.341207 | SAMN11131484 | 20.40 | -38.61 | 7054061 | 694824 | 9.85 | 14.40 | 379.47 |
| Lles_AMT22_25_04 | RMNH.MOL.340258 | SAMN11131485 | 20.40 | -38.61 | 2998157 | 468110 | 15.61 | 11.98 | 269.37 |
| Lles_AMT22_49_01 | RMNH.MOL.340259 | SAMN11131486 | -15.30 | -25.07 | 6755374 | 886610 | 13.12 | 15.59 | 485.75 |
| Lles_AMT22_49_03 | RMNH.MOL.340260 | SAMN11131487 | -15.30 | -25.07 | 6942361 | 718982 | 10.36 | 11.03 | 414.07 |
| Lles_AMT22_49_04 | RMNH.MOL.340261 | SAMN11131488 | -15.30 | -25.07 | 6789274 | 684160 | 10.08 | 10.12 | 405.38 |
| Lles_AMT22_55_02 | RMNH.MOL.340262 | SAMN11131489 | -24.13 | -25.00 | 9641976 | 988170 | 10.25 | 14.77 | 522.79 |
| Lles_AMT22_55_03 | RMNH.MOL.340263 | SAMN11131490 | -24.13 | -25.00 | 8761780 | 1139976 | 13.01 | 15.53 | 449.81 |
| Lles_AMT22_55_04 | RMNH.MOL.340264 | SAMN11131491 | -24.13 | -25.00 | 9170668 | 787034 | 8.58 | 13.31 | 448.47 |
| Lhel_AMT24_27_02 | RMNH.MOL.340265 | SAMN11131492 | -40.12 | -30.92 | 12497407 | 486658 | 3.89 | 14.18 | 95.55 |
| Lhel_AMT24_27_03 | RMNH.MOL.340266 | SAMN11131493 | -40.12 | -30.92 | 9928760 | 398788 | 4.02 | 13.50 | 71.00 |
| Lhel_AMT24_27_04 | RMNH.MOL.340267 | SAMN11131494 | -40.12 | -30.92 | 12041919 | 365014 | 3.03 | 14.66 | 65.82 |
| Lhel_AMT24_28_03 | RMNH.MOL.340268 | SAMN11131495 | -41.48 | -33.87 | 21381584 | 621646 | 2.91 | 16.72 | 105.40 |
| Lhel_AMT24_28_04 | RMNH.MOL.340269 | SAMN11131496 | -41.48 | -33.87 | 12948171 | 391250 | 3.02 | 13.51 | 69.07 |
| Lhel_AMT24_28_05 | RMNH.MOL.340270 | SAMN11131497 | -41.48 | -33.87 | 14792208 | 407548 | 2.76 | 12.24 | 64.54 |
| Lhel_CCE_123_02 | RMNH.MOL.340271 | SAMN11131498 | 48.36 | -126.31 | 4010923 | 165092 | 4.12 | 10.81 | 43.32 |
| Lhel_CCE_123_03 | RMNH.MOL.340272 | SAMN11131499 | 48.36 | -126.31 | 3787224 | 129550 | 3.42 | 9.26 | 35.30 |
| Lhel_CCE_123_06 | RMNH.MOL.340273 | SAMN11131500 | 48.36 | -126.31 | 1721637 | 70278 | 4.08 | 8.23 | 23.13 |
| Hinf_AMT22_21_02 | RMNH.MOL.340283 | SAMN11131510 | 25.48 | -39.00 | 3890761 | 92610 | 2.38 | 9.99 | 40.60 |
| Hinf_AMT22_21_03 | RMNH.MOL.340284 | SAMN11131511 | 25.48 | -39.00 | 3469925 | 55336 | 1.59 | 6.16 | 22.73 |
| Hinf_AMT22_21_06 | RMNH.MOL.340285 | SAMN11131512 | 25.48 | -39.00 | 1609269 | 31112 | 1.93 | 5.13 | 23.76 |
| Hinf_AMT22_45_01 | RMNH.MOL.340286 | SAMN11131513 | -8.08 | -25.04 | 3726617 | 77294 | 2.07 | 12.80 | 34.87 |
| Hinf_AMT22_45_02 | RMNH.MOL.340287 | SAMN11131514 | -8.08 | -25.04 | 3920742 | 91946 | 2.35 | 12.58 | 55.57 |
| Hinf_AMT22_45_03 | RMNH.MOL.340288 | SAMN11131515 | -8.08 | -25.04 | 1256559 | 24332 | 1.94 | 4.16 | 13.01 |
| Hinf_AMT22_66_01 | RMNH.MOL.340289 | SAMN11131516 | -38.08 | -39.31 | 4229048 | 107418 | 2.54 | 10.85 | 52.23 |
| Hinf_AMT22_66_02 | RMNH.MOL.340290 | SAMN11131517 | -38.08 | -39.31 | 3653019 | 71554 | 1.96 | 6.28 | 22.93 |
| Hinf_AMT22_66_04 | RMNH.MOL.340291 | SAMN11131518 | -38.08 | -39.31 | 2042221 | 38566 | 1.89 | 5.89 | 20.99 |
